# Identification of STAT5B as a biomarker associated with prognosis and immune infiltration in breast cancer

**DOI:** 10.1097/MD.0000000000032972

**Published:** 2023-03-03

**Authors:** Jiaying Li, Li Li, Gulijiang Mahesutihan, Juanjuan Meng, Yuan Chen, Jingsen Lv

**Affiliations:** a Department of Pharmacy, Branch of the First Affiliated Hospital of Xinjiang Medical University, Changji, Xinjiang, China; b Department of Party and government, Branch of the First Affiliated Hospital of Xinjiang Medical University, Changji, Xinjiang, China; c Department of Oncology, Branch of the First Affiliated Hospital of Xinjiang Medical University, Changji, Xinjiang, China; d Department of Information, Changji People’s Hospital, Changji, Xinjiang, China; e Forevergen Biosciences Center, Guangzhou, Guangdong, China.

**Keywords:** biomarkers, breast neoplasms, immune infiltration, prognosis, STAT5

## Abstract

Breast invasive cancer (BRCA) is the most common malignancy and the second leading cause of malignancy death among women. Signal transducers and activators of transcription (STAT) family played a vital role in regulating certain biological processes and could serve as biomarkers for many diseases or cancers.

**Methods:**

The expression, prognostic value, and clinical functions of STAT family in BRCA were evaluated with several bioinformatics web portals.

**Results:**

The expression of STAT5A/5B were downregulated in BRCA in subgroup analyses based on race, age, gender, race, subclasses, tumor histology, menopause status, nodal metastasis status, and TP53 mutation. BRCA patients with high STAT5B expression had a better overall survival, relapse free survival, MDFS and post progression survival. STAT5B expression level can impact the prognosis in BRCA patients with positive PR status, negative Her2 status and wild type TP53. Moreover, STAT5B was positively correlated with immune cell infiltration and the level of immune biomarkers. Drug sensitivity revealed that low STAT5B expression was resistant to the many small molecules or drugs. Functional enrichment analysis revealed that STAT5B was involved in adaptive immune response, translational initiation, JAK-STAT signaling pathway, Ribosome, NF-kappa B signaling pathway and Cell adhesion molecules.

**Conclusions:**

STAT5B was a biomarker associated with prognosis and immune infiltration in breast cancer.

## 1. Introduction

Breast invasive cancer (BRCA) is the most common malignancy and the second leading cause of malignancy death among women.^[1]^ About 1.7 million people were estimated to be diagnosed with breast cancer worldwide and 0.5 million patients were estimated to die from it.^[2,3]^ In the past of few years, treatment for breast cancer has been transferred from the original conventional surgery to conventional surgery combined with adjuvant therapy, endocrine therapy, immunotherapy, and chemotherapy.^[4]^ Therefore, great progress has been made in the prognosis of breast cancer. However, the overall prognosis of breast cancer patients was poor, especially triple negative breast cancer patients and patients with metastatic disease.^[5,6]^ Thus, these sobering data illustrate novel biomarkers for prognosis, drug screening and therapy for invasive breast cancer, providing more strategies for prognosis predicting and the optimized treatment.

Signal transducers and activators of transcription (STATs) is a family of transcription factors mediating cellular responses to cytokines and growth factors.^[7]^ Seven members of STAT family have been identified in human beings, including STAT1/2/3/4/5A/5B/6. STAT associated signaling play a vital role in regulating certain biological processes, including proliferation, metastasis, inflammation, and immune response.^[8]^ Accumulating studies revealed that STATs could serve as biomarker for many diseases or cancers, such as STAT1 for pancreatic cancer,^[9]^ STAT3 for triple negative breast cancer,^[10]^ STAT4 for gastric cancer. Though certain studies about STAT family in breast cancer have been performed, the role of STAT family was far from fully clarified.^[11]^

In our study, a comprehensive study about the expression, and prognosis significance of STAT family in breast cancer was constructed. Moreover, we also evaluate the correlation between STAT family and immune infiltration, as well as drug sensitivity. Our study may provide more serviceable information on the function of STAT family in breast cancer.

## 2. Materials and methods

### 2.1. Oncomine

Oncomine is a comprehensive platform for gene expression, and networks analysis, containing 715 datasets of 86,733 samples. The mRNA level of STAT family in BRCA were explored using Oncomine with a *P* value of 1E to 4 and a fold-change (FC) of 2.

### 2.2. GEPIA

GEPIA is a comprehensive platform for analyzing the mRNA expression data of The Cancer Genome Atlas Program (TCGA) cancers. TCGA is a landmark cancer genomics program, molecularly characterized over 20,000 primary cancer and matched normal samples spanning 33 cancer types. The difference of the expression STAT family in BRCA were evaluated using Student *t* test with TCGA STAD dataset and a *P* value <.05 was set as the threshold.

### 2.3. UALCAN

Integrating the dataset from TCGA and clinical proteomic tumor analysis consortium, UALCAN could be used to perform gene expression, prognosis analysis and other analyses.^[12]^ The correlation between STAT5A/5B expression and race, age, gender, race, subclasses, tumor histology, menopause status, nodal metastasis status, and TP53 mutation were explored with TCGA STAD dataset (n = 415) with a *P* value of 0.05 indicating statistical significance.

### 2.4. The Kaplan–Meier plotter (KM plotter)

KM plotter is designed to perform prognostic analysis for breast cancer, lung cancer, and gastric cancer. The overall survival (OS), post progression survival (PPS) and relapse free survival (RFS) and distant metastasis free survival (DMFS) analyses of STAT5B in breast cancer were performed with Kaplan–Meier curve in KM plotter. The medium value of STAT5B expression were used to split patients into high/low expression group.

### 2.5. cBioportal

cBioportal is a cancer genomics portal could be used to perform multidimensional cancer genomics analysis. Genetic alteration of STAT family, and the effect of genetic alteration of STAT family on patients’ prognosis were analyzed in cBioportal with TCGA STAD dataset. And mRNA expression (RNA Seq V2 RSEM) and protein expression clinical proteomic tumor analysis consortium were obtained using a z score threshold of ± 2.0.

### 2.6. GSCA Lite

GSCA Lite is a cancer genomics portal for gene set cancer analysis. In order to analyze the correlation between STAT5B and drug sensitivity, we collected 481 small molecules or drugs from therapeutics response portal and 265 small molecules or drugs from Genomics of Drug Sensitivity in Cancer (GDSC). Pearson correlation were used to calculate the coefficients and the significance.

### 2.7. TIMER

TIMER is designed to perform systematical analysis of immune infiltrates across diverse cancer types. The correlation between STAT5B expression and the abundance of immune cell infiltrates and the expression of gene biomarkers of immune cells were analyzed with spearman correlation.^[13–15]^ Two-sided Wilcoxon rank-sum test was used to evaluate the role of STAT5B somatic copy number alterations (SCNA) on immune cell infiltrates.

### 2.8. Linked omics

Linked omics is designed to perform evaluate, analyze and compare cancer multi-omics data using TCGA datasets. In current study, spearman correlation test was used to explore STAT5B-associated genes in BRCA according to the TCGA BRCA dataset. This is followed by Gene Set Enrichment Analysis (GSEA), which could perform GO analysis, KEGG pathways, Kinase target, miRNA target, and transcription factor-target analysis of STAT5B in BRCA. The minimum number of genes were set as 3 and *P* value < .05 indicates statistical significance.

## 3. Results

### 3.1. The expression of STAT5A and STAT5B were downregulated in BRCA.

**Figure 1.**
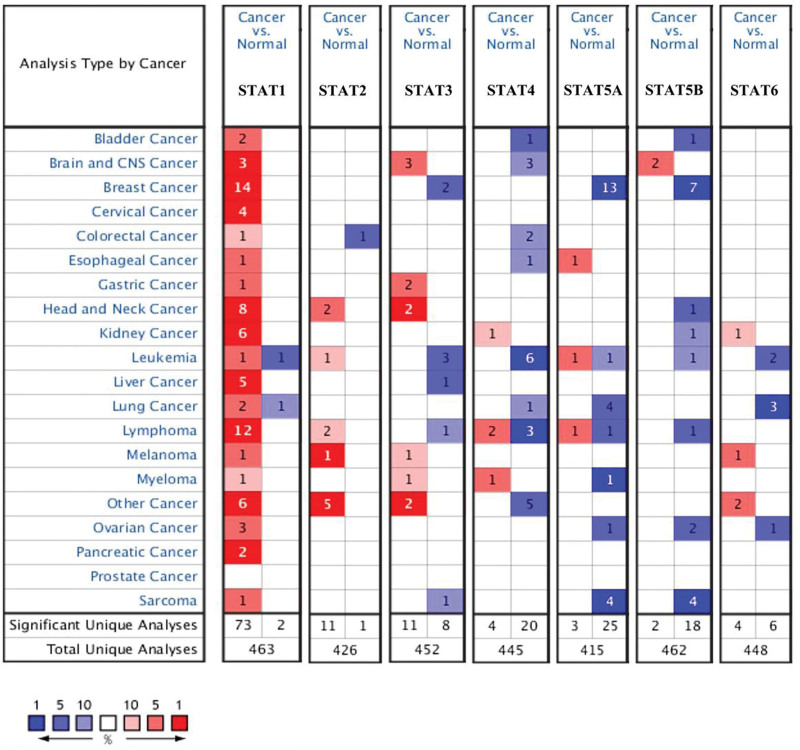
STAT family expression in BRCA at mRNA level (Oncomine). The number in the figure was the numbers of datasets with statistically significant (*P* < .01) mRNA overexpression (red) or down-expression (blue) of STAT family, which was obtain with the *P* value of 1E–4 and fold change of 2. BRCA = breast invasive cancer, STAT = signal transducers and activators of transcription.

**Figure 2.**
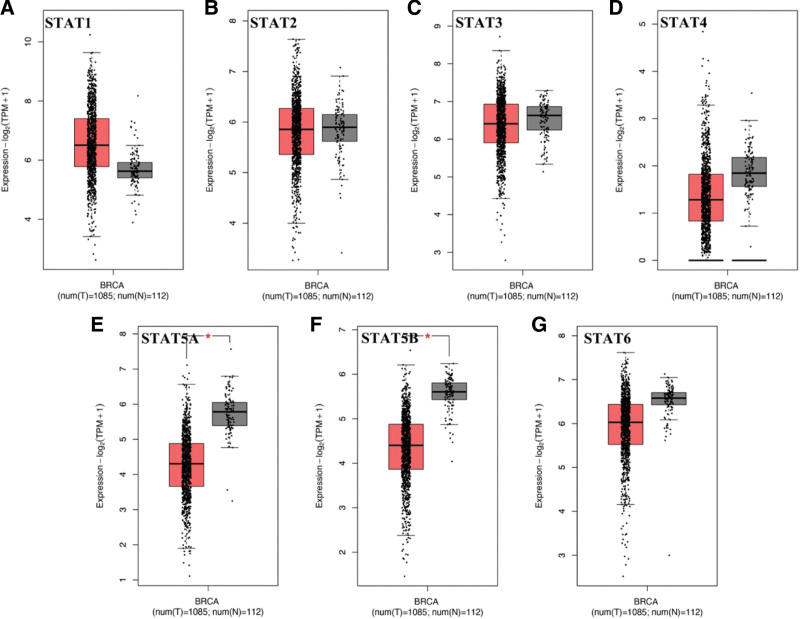
STAT family expression in BRCA at mRNA level (GEPIA). Box plots derived from gene expression data for GEPIA comparing the expression of a specific STAT family member in BRCA tissue and normal tissues; the *P* value was set at 0.05. *Indicate that the results are statistically significant. BRCA = breast invasive cancer, STAT = signal transducers and activators of transcription.

According to the results of oncomine, the expression of STAT1 was increased and the expression of STAT3/5A/5B were decreased in tumor tissues compared with normal tissues (Fig. 1 and Table 1). Two datasets indicated an increasing level of STAT1 in tumor tissues with a FC of 3.207 and 3.209, respectively (all, *P* < .05). Greg et al suggested that STAT3 was downregulated in invasive breast carcinoma and a FC was -11.013 (*P* = 1.21E–15). The result of 3 datasets demonstrated that STAT5A was downregulated in BRCA tissues, and the FCs were −2.675, −2.134, and −5.644, respectively (all, *P* < .05). Moreover, STAT5B expression in invasive ductal breast carcinoma was significantly lower than normal breast cancer (FC = −2.087, *P* = 4.17E–79). Another dataset also suggested a downregulation of STAT5B expression in invasive ductal and lobular breast carcinoma (FC = −2.067, *P* = 2.51E*–*36). We then verified our result in GEPIA using TCGA BRCA dataset. As a result, the level of STAT5A (Fig. 2E) and STAT5B (Fig. 2F) were significantly downregulated in tumor tissues compared with normal tissues (*P* < .05). However, there is no significant difference in the expression of STAT1 (Fig. 2A), STAT2 (Fig. 2B), STAT3 (Fig. 2C), STAT4 (Fig. 2D), and STAT6 (Fig. 2G) between tumor tissues and normal tissues. Combined with above results, the expression of STAT5A and STAT5B were downregulated in BRCA.

**Table 1:** The mRNA levels of STAT family in BRCA (ONCOMINE).

TLR	type	Fold Change	*P* value	*t* test	Reference
STAT1	invasive breast carcinoma	3.207	1.06E–7	7.389	PMID:22522925
	invasive ductal breast carcinoma	3.209	3.48E–5	7.638	PMID:15034139
STAT2	NA	NA	NA	NA	NA
STAT3	invasive breast carcinoma	−11.013	1.21E–15	−17.246	PMID:18438415
STAT4	NA	NA	NA	NA	NA
STAT5A	invasive breast carcinoma	−2.675	1.32E–11	−11.063	PMID:22522925
	invasive ductal breast carcinoma	−2.134	7.70E–7	−7.766	PMID:15034139
	invasive breast carcinoma	−5.644	1.91E–23	−18.355	PMID:18438415
STAT5B	invasive ductal breast carcinoma	−2.087	4.17E–79	−32.416	PMID:22522925
	invasive ductal and lobular breast carcinoma	−2.067	2.51E–36	−16.849	PMID:22522925
STAT6	NA	NA	NA	NA	NA

STAT = signal transducers and activators of transcription.

**Figure 3.**
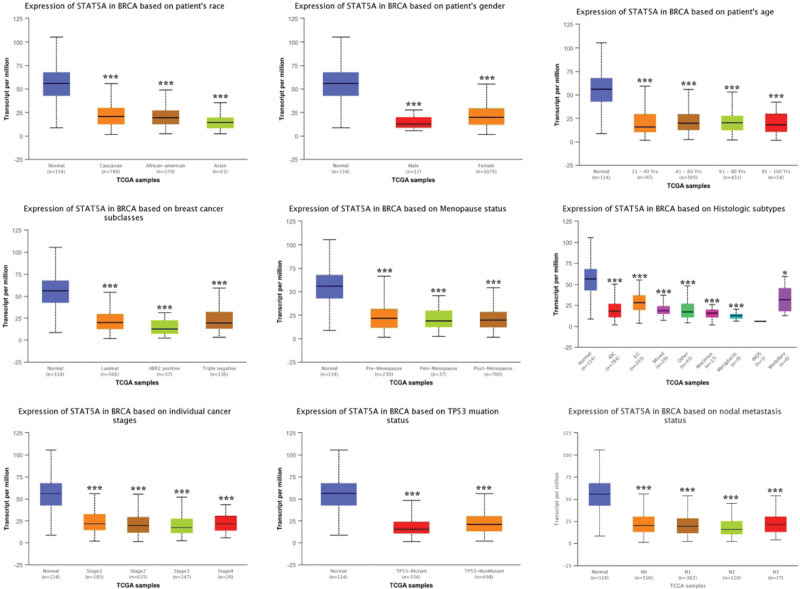
STAT5A expression in subgroups of patients with BRCA (UALCAN). Boxplot showing relative expression of STAT5A in normal and BRCA patients in subgroup analyses based on race, age, gender, race, subclasses, tumor histology, menopause status, nodal metastasis status, and TP53 mutation. Data are mean ± SE. *P < .05; †*P* < .001. BRCA = breast invasive cancer, STAT = signal transducers and activators of transcription.

**Figure 4.**
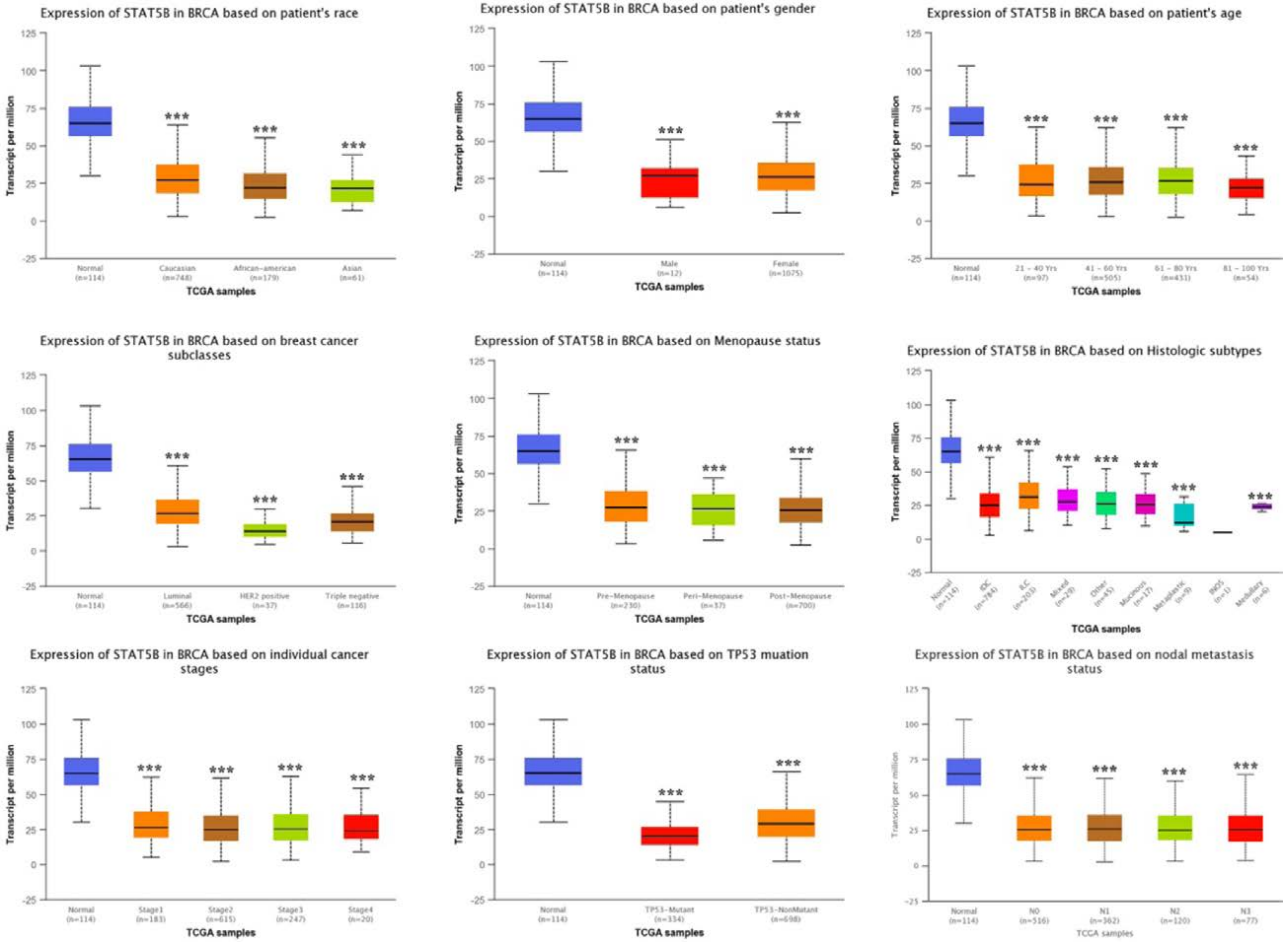
STAT5B expression in subgroups of patients with BRCA (UALCAN). Boxplot showing relative expression of STAT5B in normal and BRCA patients in subgroup analyses based on race, age, gender, race, subclasses, tumor histology, menopause status, nodal metastasis status, and TP53 mutation. Data are mean ± SE. *P < .001. BRCA = breast invasive cancer, STAT = signal transducers and activators of transcription.

We then performed subgroup analysis and evaluated the correlation between the level of STAT5A/5B and clinic pathological features. As expected, the result found that STAT5A expression was remarkably downregulated in BRCA compared with healthy control in subgroup analyses based on race, age, gender, race, subclasses, tumor histology, menopause status, nodal metastasis status, and TP53 mutation (Fig. 3). Similar results were obtained in STAT5B. STAT5B expression was significantly downregulated in BRCA compared with healthy control in subgroup analyses based on race, age, gender, race, subclasses, tumor histology, menopause status, nodal metastasis status and TP53 mutation (Fig. 4). Thus, STAT5A and STAT5B may help to detect invasive breast cancer patients.

### 3.2. Co-expression, genetic alteration and drug sensitivity of STAT family in BRCA

**Figure 5.**
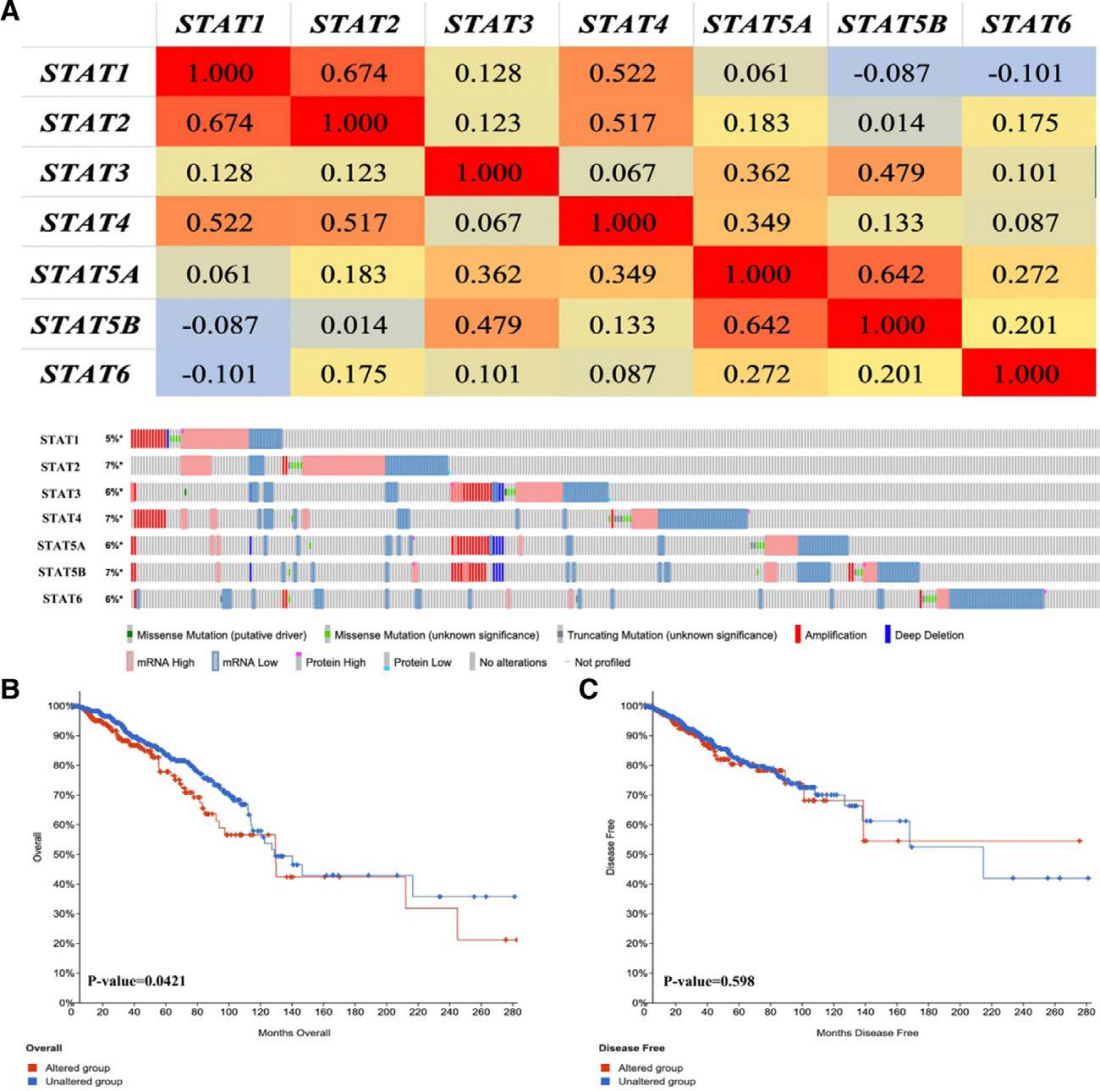
Co-expression, Genetic alteration analyses of STAT family in BRCA patients (cbioportal). (A) Correlation heat map of STAT family in BRCA. (B) Summary of genetic alterations in STAT family in BRCA. (C) Kaplan–Meier plots comparing overall survival in cases with/without STAT family genetic alterations. (D) Kaplan–Meier plots comparing disease-free survival in cases with/without STAT family genetic alterations. BRCA = breast invasive cancer, STAT = signal transducers and activators of transcription.

**Figure 6.**
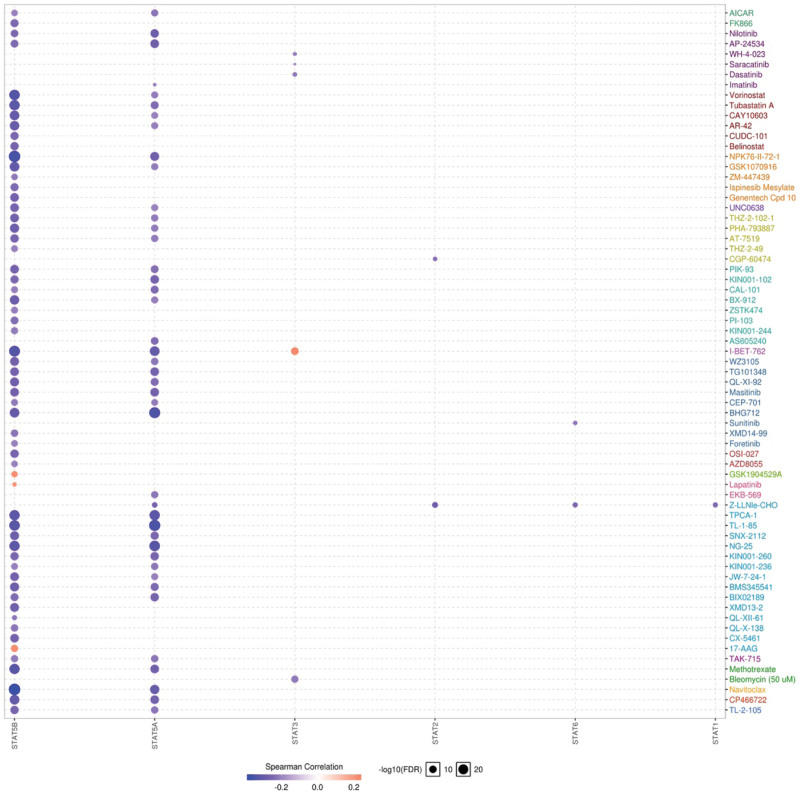
The drug resistance analysis of STAT family based on GDSC IC50 drug data. The Spearman correlation represent the gene expression correlates with the drug. The positive correlation means that the gene high expression is resistant to the drug, vise verse. BRCA = breast invasive cancer, GDSC = genomics of drug sensitivity in cancer, STAT = signal transducers and activators of transcription.

We then performed co expression and genetic alteration of STAT family in BRCA. We found a high correlation among the expression of STAT1 and STAT2, as well as STAT5A and STAT5B (Fig. 5A). Moreover, a low to moderate correlation was obtained among STAT3, STAT4 and STAT6 (Fig. 5A). Genetic alteration analysis revealed that STAT1, STAT2, STAT3, STAT4, STAT5A, STAT5B and STAT6 were altered in 5%, 7%, 6%, 7%, 6%, 7% and 6% of the queried BRCA samples, respectively (Fig. 5B). Genetic alteration of STAT family in BRCA include missense mutation, truncating mutation, amplification, deep deletion, mRNA high, mRNA low, protein high and protein low. Moreover, we found that genetic alteration of STAT family in BRCA could affect the overall survival (Fig. 5C, *P* = .0421) but not disease-free survival (Fig. 5D, *P* = .598) of patients. Pearson correlation was performed to analyze the correlation between STAT family and 265 small molecules or drugs from GDSC. As a result, low STAT5B and STAT5A expression were resistant to the 57 and 48 small molecules or drugs of GDSC, respectively (Fig. 6). Therefore, STAT5A and STAT5B may act as the biomarkers for drug screening.

### 3.3. STAT5B served as a prognostic biomarker in BRCA

**Figure 7.**
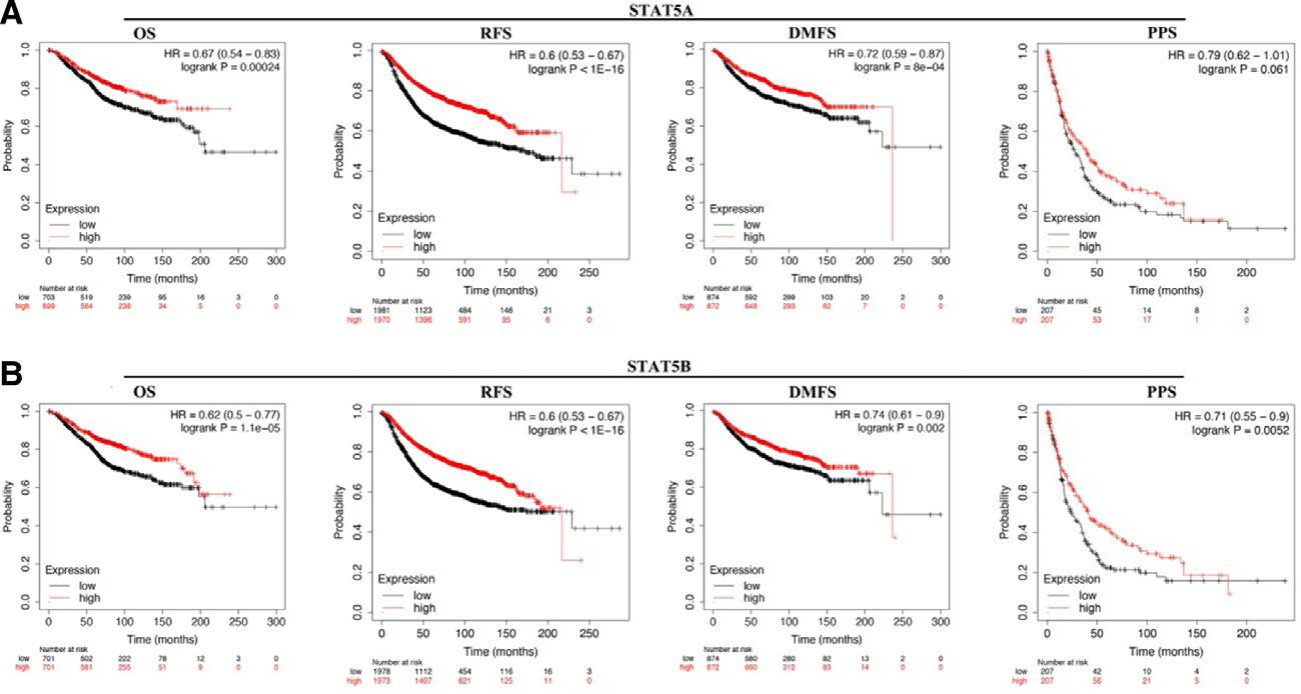
The prognostic value of STAT5A/5B in BRCA. (A) The OS, RFS, DMFS, and PPS curve in BRCA patients with high and low expression of STAT5A. (B) The OS, RFS, DMFS, and PPS curve in BRCA patients with high and low expression of STAT5B. BRCA = breast invasive cancer, DMFS = distant metastasis free survival, OS = overall survival, PPS = post progression survival, RFS = relapse free survival, STAT = signal transducers and activators of transcription.

STAT5A and STAT5B were selected for prognostic analysis in BRCA. As shown in Figure 7A, BRCA patients with high STAT5A expression had a better OS (HR = 0.67 (0.54–0.83), *P* = .00024), RFS (HR = 0.6 (0.53–0.67), *P* < 1E–16), MDFS (HR = 0.72 (0.59–0.83), *P* = 8E–4). However, STAT5A expression could not affect the PPS of BRCA patients (*P* = .061). Moreover, BRCA patients with high STAT5B expression had a better OS (HR = 0.62 (0.5–0.77), *P* = 1.1E–5), RFS (HR = 0.6 (0.53–0.67), *P* < 1E–16), MDFS (HR = 0.74 (0.61–0.9), *P* = .002) and PPS (HR = 0.71 (0.55–0.9), *P* = .0052) (Fig. 7B). Therefore, STAT5B served as a prognostic biomarker in BRCA.

In order to better clarify how STAT5B expression affect the prognosis of BRCA patients, we further explore the relationship between the STAT5B expression and clinical characteristics. We found that upregulation of STAT5B was associated with better OS and RFS in BRCA patients in positive and negative ER status (Table 2, *P* < .05). Besides, upregulation of STAT5B was associated with better OS, RFS, DMFS, and PPS in BRCA patients in positive and negative lymph node status (Table 2 and Table, *P* < .05). Interestingly, high STAT5B expression was correlated with better OS, RFS, and DMFS in positive PR status BRCA patients (Table 2 and Table 3) but not negative PR status BRCA patients (Table 3). Moreover, high STAT5B expression was correlated with better OS, RFS and DMFS in negative Her2 status BRCA patients (Table 2 and Table 3) but not positive Her2 status BRCA patients (Table 3). We also found that high STAT5B expression was correlated with better OS, DMFS and PPS in wild type TP53 BRCA patients (Table 2 and Table 3) but not mutated TP53 BRCA patients (Table 3). Therefore, these results demonstrated that STAT5B expression level can impact the prognosis in BRCA patient with positive PR status, negative Her2 status and wild type TP53.

**Table 2:** Correlation of STAT5B mRNA expression and overall survival in BRCA with different clinicopathological factors (Kaplan–Meier plotter).

Pathological parameters	OS			RFS		
	N	Hazard radio	*P* value	N	Hazard radio	*P* value
ER status						
Positive	548	0.62 (0.44–0.89)	.0078	2061	0.7 (0.59–0.83)	3.4e^-5^
Negative	251	0.52 (0.28–0.97)	.037	801	0.7 (0.53–0.93)	.012
PR status						
Positive	83	0.29 (0.06–1.38)	.096	589	0.71 (0.49–1.03)	.073
Negative	89	2.3 (0.76–7)	.13	549	0.64 (0.45–0.91)	.011
HER2 status						
Positive	129	1.46 (0.71–3)	.3	252	0.58 (0.37–0.9)	.015
Negative	130	0 (0–lnf)	.017	800	0.63 (0.49–0.82)	.00057
Intrinsic subtype						
Basal	241	0.79 (0.47–1.33)	.36	618	0.64 (0.5–0.84)	.00086
Luminal A	611	0.6 (0.41–0.87)	.0059	1933	0.54 (0.45–0.65)	4.1e-^12^
Luminal B	433	0.53 (0.36–0.76)	.00056	1149	0.59 (0.49–0.72)	6.1e^-8^
Lymph node status						
Positive	313	0.56 (0.37–0.87)	.008	1133	0.66 (0.54–0.8)	3e^-5^
Negative	594	0.46 (0.26–0.79)	.0039	2020	0.83 (0.7–0.99)	.036
Tumor grade						
1	161	0.42 (0.14–1.3)	.12	345	0.7 (0.4–1.23)	.21
2	387	0.47 (0.3–0.72)	.00043	901	0.72 (0.57–0.92)	.008
3	503	0.66 (0.45–0.97)	.035	903	0.83 (0.66–1.04)	.11
TP53 status						
Mutated	111	2.26 (0.67–7.61)	.18	188	0.62 (0.37–1.02)	.055
Wild type	187	0.32 (0.16–0.64)	.00065	273	0.76 (0.5–1.18)	.22

OS = overall survival, RFS = relapse free survival, STAT = signal transducers and activators of transcription.

**Table 3:** Correlation of STAT5B mRNA expression and overall survival in BRCA with different clinicopathological factors (Kaplan–Meier plotter).

Pathological parameters	DMFS				PPS		
	N	Hazard radio	*P* value	N		Hazard radio	*P* value
ER status							
Positive	664	0.7 (0.46–1.08)	.1	173		0.77 (0.5–1.19)	.24
Negative	218	0.62 (0.35–1.12)	.11	100		0.45 (0.22–0.95)	.032
PR status							
Positive	192	3.79 (1.28–11.24)	.0098	13			
Negative	154	0.69 (0.36–1.34)	.28	17			
HER2 status							
Positive	126	1.34 (0.69–2.58)	.39	33		1.66 (0.53–5.14)	.38
Negative	150	0.29 (0.09–0.99)	.035	39		0.43 (0.16–1.21)	.1
Intrinsic subtype							
Basal	232	0.66 (0.39–1.1)	.11	64		1.44 (0.8–2.6)	.22
Luminal A	965	0.8 (0.6–1.06)	.12	179		0.68 (0.46–1)	.048
Luminal B	430	0.65 (0.46–0.92)	.015	134		0.66 (0.43–1.01)	.056
Lymph node status							
Positive	382	0.54 (0.35–0.82)	.0033	128		1.48 (0.94–2.32)	.088
Negative	988	0.68 (0.51–0.89)	.0057	165		0.47 (0.31–0.74)	.00069
Tumor grade							
1	188	0.68 (0.29–1.61)	.38	34		0.28 (0.11–0.77)	.0081
2	546	0.74 (0.52–1.05)	.09	128		0.74 (0.46–1.2)	.22
3	458	1.2 (0.85–1.7)	.3	165		0.58 (0.36–0.95)	.029
TP53 status							
Mutated	83	0.42 (0.14–1.28)	.12	34		0.65 (0.27–1.6)	.35
Wild type	109	0.34 (0.15–0.79)	.0089	62		0.43 (0.21–0.85)	.013

DMFS = distant metastasis free survival, PPS = post progression survival, STAT = signal transducers and activators of transcription.

### 3.4. Predictive nomogram

**Figure 8.**
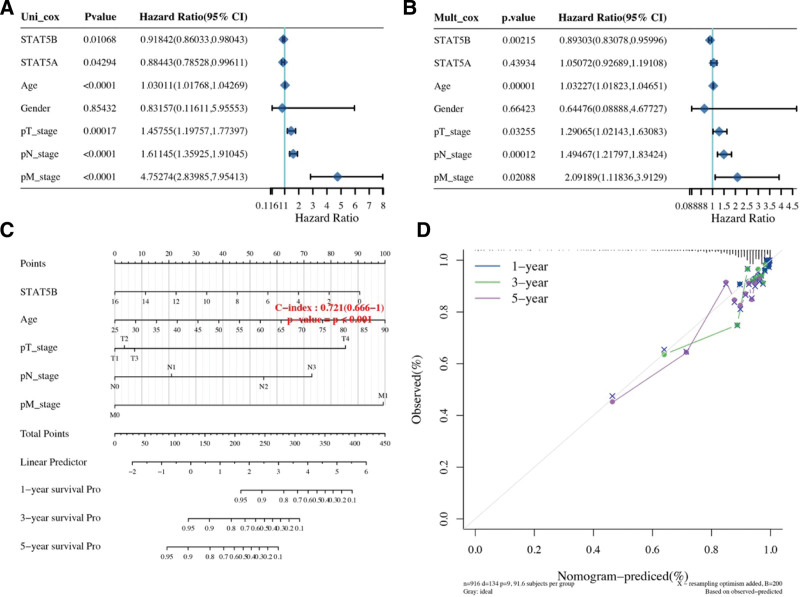
Univariate and multivariate cox regression of STAT5A/5B in BRCA. (A–B) Univariate and multivariate cox regression considering patients’ parameters and STAT5A/5B. (C–D) Nomogram to predict the1-y, 3-y and 5-y overall survival of breast cancer patients. Calibration curve for the overall survival nomogram model in the discovery group. A dashed diagonal line represents the ideal nomogram.

Considering clinicopathologic features, STAT5A and STAT5B, we developed a predictive nomogram to construct a predictive model. As shown in Figure 8A and B, the univariate and multivariate analysis revealed that STAT5B, age, pT stage, pN stage and pM stage were independent factors affecting the prognosis of breast cancer patients. Based on cox regression algorithm, a predictive nomogram was constructed, indicating that the calibration plots for the 5-year OS rates were predicted relatively well compared with an ideal model in the entire cohort (Fig. 8C–D).

### 3.5. STAT5B correlated with immune infiltrates in BRCA.

**Figure 9.**
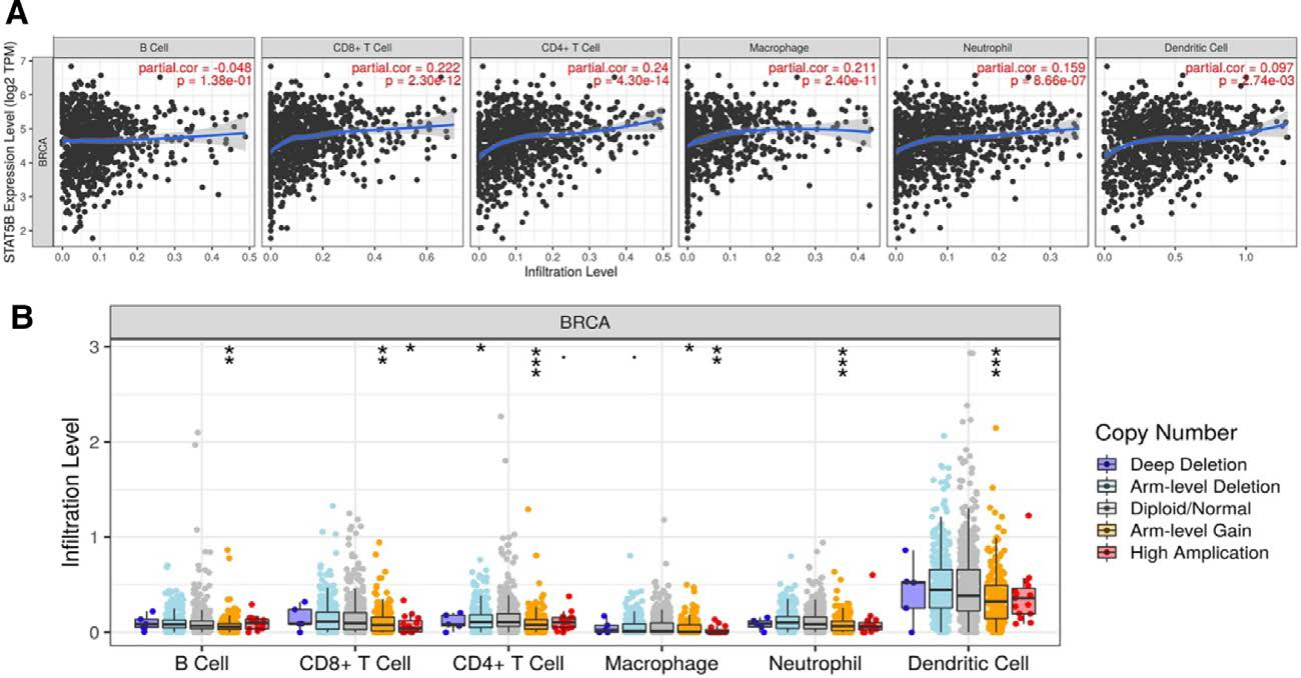
Immune infiltration STAT family in BRCA. (A) The correlation between STAT5B and the abundance of different immune cell level in BRCA. (B) The correlation between copy number alteration of STAT5b and immune cell infiltration in BRCA. *P < .05; †*P* < .01, ‡*P* < .001. BRCA = breast invasive cancer, STAT = signal transducers and activators of transcription.

Previous study revealed that STAT family were involved in inflammation and immune response. Thus, we analyzed correlation between STAT5B and immune infiltrates in BRCA. The results revealed that STAT5B expression showed a positive correlation between immune infiltrates level of CD8 + T cells (Cor = 0.222, *P* = 2.30e–12), CD4 + T cells (Cor = 0.24, *P* = 4.3e–14), Macrophage (Cor = 0.211, *P* = 2.4e–11), Neutrophil (Cor = 0.159, *P* = 8.66e–7) and Dendritic cells (Cor = 0.097, *P* = 2.74e–03) (Fig. 9A). Interestingly, we also found that STAT5B SCNA could partially inhibit immune infiltrates level in BRCA (Fig. 9B).

Moreover, immune gene biomarkers analysis also demonstrated a significant association between STAT5B and gene biomarkers in BRCA (Table 4). Specifically, for the gene biomarkers of CD8 + T cell (D8A, CD8B), T cell (CD3D, CD3E, CD2), B cell (CD19, CD79A), Monocyte (CD115), and TAM (CCL2, IL10), M1 Macrophage (NOS2, IRF5), and *M*2 Macrophage (CD163, VSIG4, and MS4A4A), their expressions were positively associated with STAT5B expression in BRCA. The level of CD11b, CCR7, KIR2DL4, and KIR3DL1 showed correlation with STAT5B level in BRCA. All the gene biomarkers of Dendritic cell were positively associated with STAT5B level in BRCA. Moreover, most gene biomarkers of Th1 (TBX21, STAT4, STAT1, TNF), Th2 (GATA3, STAT6, STAT5A) and Tfh (BCL6) show positive association with STAT5B level in BRCA. We also revealed the remarkable correlation in between the expression of gene biomarkers (LAG3, TIM-3, GZMB) of T cell exhaustion and STAT5B. Therefore, STAT5B correlated with immune infiltrates and may play a significant role in tumor immune escape in BRCA.

**Table 4:** Correlation analysis between STAT5B and gene biomarkers of immune cells in BRCA (TIMER).

Description	Biomarkers	STAT5B	
		Cor	*P* value
CD8 + T cell	CD8A	0.168	***
	CD8B	0.1	***
T cell (general)	CD3D	0.063	*
	CD3E	0.11	***
	CD2	0.085	**
B cell	CD19	0.076	*
	CD79A	0.082	**
Monocyte	CD86	0.099	**
	CD115(CSF1R)	0.256	***
TAM	CCL2	0.105	***
	CD68	0.081	**
	IL10	0.113	***
M1 macrophage	INOS (NOS2)	0.092	**
	IRF5	0.184	***
	COX2(PTGS2)	0.213	***
M2 macrophage	CD163	0.118	***
	VSIG4	0.142	***
	MS4A4A	0.124	***
Neutrophils	CD66b (CEACAM8)	0.008	0.793
	CD11b (ITGAM)	0.199	***
	CCR7	0.163	***
Natural killer cell	KIR2DL1	0.029	0.33
	KIR2DL3	0.009	0.769
	KIR2DL4	0.06	*
	KIR3DL1	0.06	*
	KIR3DL2	0.027	0.371
	KIR3DL3	−0.01	0.732
	KIR2DS4	−0.025	0.413
Dendritic cell	HLA-DPB1	0.14	***
	HLA-DQB1	0.076	*
	HLA-DRA	0.157	***
	HLA-DPA1	0.199	***
	BDCA-1(CD1C)	0.269	***
	BDCA-4(NRP1)	0.315	***
	CD11c (ITGAX)	0.149	***
Th1	T-bet (TBX21)	0.097	**
	STAT4	0.198	***
	STAT1	0.088	**
	IFN-g (IFNG)	−0.011	0.722
	TNF-a (TNF)	0.114	***
Th2	GATA3	0.266	***
	STAT6	0.0.398	***
	STAT5A	0.693	***
	IL13	0.046	0.124
Tfh	BCL6	0.283	***
	IL21	0.047	0.119
Th17	STAT3	0.627	***
	IL17A	0.001	0.972
Treg	FOXP3	0.02	0.518
	CCR8	0.115	***
	STAT5B	NA	NA
	TGFb (TGFB1)	0.212	***
T cell exhaustion	PD-1 (PDCD1)	0.024	0.428
	CTLA4	−0.011	0.728
	LAG3	−0.167	***
	TIM-3 (HAVCR2)	0.0.108	***
	GZMB	−0.062	*

STAT = signal transducers and activators of transcription.

* *P* < .05,

** *P* < .01,

*** *P* < .001.

### 3.6. Enrichment analysis of STAT5B in BRCA.

**Figure 10.**
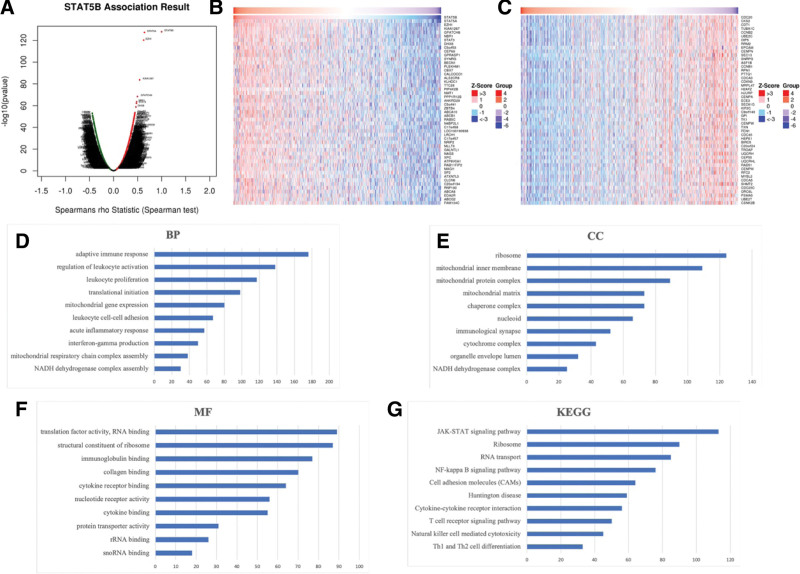
The enrichment analysis of STAT5B in BRCA. (A) A Pearson test was used to analyze correlations between STAT5B and genes differentially expressed in BRCA. (B–C) Heat maps showing genes positively and negatively correlated with STAT5B in BRCA (TOP 50). Red indicates those genes positively correlated with STAT5B and green indicates those genes negatively correlated with STAT5B. (D–F) Heatmap of GO enrichment in CC terms, BP terms and MF terms. (G) KEGG pathways analysis. GO and KEGG were performed by Gene Set Enrichment Analysis. BP = biological process, BRCA = breast invasive cancer, CC = molecular function, GO = gene ontology, KEGG = Kyoto encyclopedia of genes and genomes, MF = molecular functions, STAT = signal transducers and activators of transcription.

We first explored the genes associated with STAT5B in BRCA. As a result, 7774 genes (0.08 < cor < 1) positively associated with STAT5B and 3768 genes (−1 < cor < −0.08) negatively associated with STAT5B in BRCA were obtained (Fig. 10A, FDR < 0.01). We also extracted the top 50 most significant genes positively and negatively associated with STAT5B in BRCA, which were shown in Figure 10B and C. The genes for GO and KEGG pathways were shown in Table S1, Supplemental Digital Content, http://links.lww.com/MD/I488. The GO items shown in Figure 10D to F indicated that STAT5B were involved in adaptive immune response, regulation of leukocyte activation, translational initiation, leukocyte cell-cell adhesion, translation factor activity, structural constituent of ribosome, cytokine receptor binding, immunoglobulin binding, and protein transporter activity. Besides, the KEGG items demonstrated that STAT5B were associated with JAK-STAT signaling pathway, Ribosome, RNA transport, NF-kappa B signaling pathway and Cell adhesion molecules (CAMs) (Fig. 10G).

## 4. Discussion

Breast cancer still poses a major threat to the health and well-being of American women, holding 30% of all new diagnostic cases and causing nearly 41,000 deaths each year.^[16]^ The overall prognosis of breast cancer patients was poor, especially triple negative breast cancer patients and patients with metastatic disease.^[17]^ Therefore, it is significant to identify innovative biomarkers for the prognosis and therapy for breast cancer.

Expression analysis revealed that the expression of STAT5A and STAT5B were downregulated in BRCA. Moreover, the expression STAT5A and STAT5B were remarkably downregulated in BRCA compared with healthy control in subgroup analyses based on race, age, gender, race, subclasses, tumor histology, menopause status, nodal metastasis status and TP53 mutation. There results demonstrated that STAT5A/5B may associated with BRCA aggression and STAT5A/5B may help to detect invasive breast cancer patients. Previous studies have suggested that STAT5A/5B could regulate the tumor cell proliferation, invasion and metastasis in breast cancer. For example, STAT5A could promote the proliferation and metastasis of breast cancer cells by mediating NOX5-L expression.^[18]^ Another study revealed that STAT5B could inhibit tumor cell invasive characteristics of BRCA.^[19]^ Jak2/STAT5B pathway was associated with inhibiting tumor growth and metastasis in ER-positive breast cancer.^[20]^

Prognosis analysis revealed that high STAT5B expression had a better OS, RFS, MDFS and PPS. STAT5B expression level can impact the prognosis in BRCA patient with positive PR status, negative Her2 status and wild type TP53. These suggested that STAT5B served as a prognostic biomarker in BRCA. Actually, STAT5B also suggested to be a prognostic biomarker in certain types of cancers. In hepatocellular carcinoma, STAT5A/5B/6 were suggested to be potential prognostic markers.^[21]^ Moreover, STAT5B/6 could be potential biomarkers for the prognosis of non-small cell lung cancer.^[22]^ Another study indicated that low STAT5B expression was associated with a poor overall survival of ovarian cancer patients.^[23]^ Moreover, STAT5B was a prognostic biomarker for chronic lymphocytic leukemia.^[24]^ Another study suggested STAT5B as prognostic biomarker for renal cell carcinoma.^[25]^

Another important finding of our study is that STAT5B level showed positive correlation with immune cells infiltrates and the level of immune biomarkers. Immune infiltrates play a significant role in tumor microenvironment.^[26]^ Besides, dysregulation of immune cells and immune gene biomarkers exert an important role in mediating tumor immune escape, which could result in tumor progression and metastasis.^[27,28]^ Besides, certain immune cells and immune gene biomarkers could be biomarkers for the prognosis or therapy of various types of cancers, including breast cancer.

The presence of CD8 + T cells in breast cancer is associated with a significant reduction in the relative risk of death from disease.^[29]^ Moreover, CD4^+^ follicular helper T cell infiltration could predict the prognosis of breast cancer patients.^[30]^ In breast cancer, high density of tumor associated macrophages could predict poor survival rates of patients.^[31]^ Therefore, STAT5B may play a significant role in tumor immune escape, and STAT5B may act as the potential biomarker for immunotherapy in breast cancer.

In order to further verify the potential of STAT5B as therapy target of breast cancer, we then analyzed the correlation between STAT5B and drug sensitivity. As a result, we found that low STAT5B expression was resistant to the many small molecules or drugs. Our study first revealed the significant correlation between STAT5B and many small molecules or drugs, such as NG-25, Nilotinib, and masitinib. In mantle cell lymphoma, STAT5B also STAT5B was associated with drug resistance.^[32]^ Another result suggested that STAT5B could be open novel therapeutic target for drug development.^[33]^ Therefore, STAT5B may act as biomarker for drug screening in breast cancer, and further study should be performed to verify this.

Enrichment analysis of STAT5B in breast cancer were also conducted. Functional enrichment analysis revealed that STAT5B was involved in adaptive immune response, translational initiation, JAK-STAT signaling pathway, Ribosome, NF-kappa B signaling pathway and Cell adhesion molecules. Previous studies had revealed that these functions and pathways were involved in immune response and the pathogenesis and progress of cancers. NF-kB family was crucial for immune responses and inflammation and NF-kB has been implicated in the initiation, progression and resistance to treatment in human cancers.^[34]^ JAK-STAT signaling pathway is essential to tumor cell proliferation, metastasis, inflammation, and immune response in breast cancer.^[35,36]^ Cell adhesion molecules also play a significant role in breast cancer cell invasion and metastasis.^[37]^ Therefore, STAT5B may affect the tumorigenesis and progress of breast cancer by mediating these signaling pathways.

## 5. Conclusions

In conclusion, we suggested STAT5B a prognosis biomarker and associated with immune infiltration in breast cancer, providing more serviceable information on the role of STAT5B in tumorigenesis.

## Author contributions

**Formal analysis:** Juanjuan Meng.

**Funding acquisition:** Gulijiang Mahesutihan.

**Investigation:** Jiaying Li, Li Li.

**Methodology:** Li Li.

**Project administration:** Jingsen Lv.

**Resources:** Yuan Chen.

**Supervision:** Gulijiang Mahesutihan, Jingsen Lv.

**Validation:** Juanjuan Meng.

**Writing – original draft:** Jiaying Li, Li Li.

**Writing – review & editing:** Gulijiang Mahesutihan, Juanjuan Meng, Yuan Chen, Jingsen Lv.

## Supplementary Material


